# Concurrent Silicosis and Pulmonary Mycosis at Death

**DOI:** 10.3201/eid1602.090824

**Published:** 2010-02

**Authors:** Yulia Iossifova, Rachel Bailey, John Wood, Kathleen Kreiss

**Affiliations:** Centers for Disease Control and Prevention, Morgantown, West Virginia, USA; 1 Current affiliation: Centers for Disease Control and Prevention, Chamblee, Georgia, USA.

**Keywords:** pneumoconioses, mycoses, silicosis, aspergillosis, coccidioidodomycosis, asbestosis, fungi, dispatch

## Abstract

To examine risk for mycosis among persons with silicosis, we examined US mortality data for 1979–2004. Persons with silicosis were more likely to die with pulmonary mycosis than were those without pneumoconiosis or those with more common pneumoconioses. Health professionals should consider enhanced risk for mycosis for silica-exposed patients.

The pneumoconioses are a group of irreversible but preventable interstitial lung diseases, most commonly associated with inhalation of asbestos fibers, coal mine dust, or crystalline silica dust. In response to inquiries from silica-exposed workers concerned about diagnoses of coccidioidomycosis or cryptococcal meningitis for their co-workers, we examined whether excess risk for mycosis exists among persons with silicosis.

## The Study

We examined mortality data from the National Center for Health Statistics of the Centers for Disease Control and Prevention, which are coded for causes of death reported on death certificates for all US residents according to the International Classification of Diseases (ICD) (www.cdc.gov/nchs/products/elec_prods/subject/mortmcd.htm). Because silicosis usually has a latency of >20 years, we restricted our analyses to persons >45 years of age at death. We separately evaluated 3 groups of persons who had died with pulmonary mycosis coded as either the underlying or the contributing cause of death (any mention on death certificate) during 1979–2004: 1) those with any mention of silicosis versus no mention; 2) those with any mention of asbestosis versus no mention; and 3) those with any mention of coal worker pneumoconiosis (CWP) versus no mention. We excluded decedents with >2 pneumoconioses.

We analyzed mortality data for persons assigned the following ICD codes (9th revision [ICD-9] 1979–1998; 10th revision [ICD-10] 1999–2004): 502/J62 (silicosis); 501/J61 (asbestosis); 500/J60 (CWP); and 110–118/B35–B49 (any mycosis). We defined pulmonary mycosis as death with ICD-9 and ICD-10 codes 112.4/B37.1 (candidiasis); 114/B38.0, B38.1, B38.2, B38.9 (coccidioidomycosis); 115/B39.0, B39.1, B39.2, B39.4, B39.9 (histoplasmosis); 116.0/B40.0, B40.1, B40.2, B40.9 (blastomycosis); 116.1/B41.0, B41.9 (paracoccidioidomycosis); 117.1/B42.0, B42.9 (sporotrichosis); 117.7/B46.0, B46.5, B46.9 (zygomycosis); 117.3/B44.0, B44.1, B44.9 (aspergillosis); 117.5/B45.0, B45.9 (cryptococcosis); and 118/B48.7 (opportunistic mycoses). For many mycoses, ICD-9 codes do not differentiate pulmonary from other types of mycoses. For ICD-10 codes, we limited data to mycoses coded as pulmonary, opportunistic, and some unspecified type of mycoses (e.g., B38.9, B39.4, B39.9, B40.9, B41.9, B42.9, B44.9, B45.9, B46.5, and B46.9). We provided results with and without ICD-9 code for opportunistic mycoses and ICD-10 codes for unspecified mycoses and opportunistic mycoses.

We computed prevalence rate ratios and 95% confidence intervals (CIs) to separately compare pulmonary mycosis prevalence at death among persons with silicosis, asbestosis, and CWP with that for persons in the referent group and to compare pulmonary mycosis prevalence at death among persons with silicosis with that for persons in the 2 pneumoconiosis comparison groups. Each ratio was computed by dividing the proportion of mycosis deaths in 1 group by the corresponding measure in the comparison group.

Decedents with pneumoconiosis and mycosis were rare, and most mycoses were pulmonary: 77% in persons with silicosis; 79% asbestosis, and 53% CWP (Table 1). Persons with silicosis were 4.5× (95% CI 3.4–6.0×) more likely to have any mycosis at death and 9.5× (95% CI 6.9–13.1×) more likely to have pulmonary mycosis at death than were those without pneumoconiosis. Persons with silicosis were 2.9× (95% CI 1.9–4.4×) more likely than those with asbestosis and 6.7× (95% CI 4.3–10.5×) more likely than those with CWP to have pulmonary mycosis at death.

**Table 1:** Cause of death with any death certificate mention of selected pneumoconioses and mycoses (underlying or contributing cause), US residents >45 y of age, 1979–2004*

Cause	No. deaths	Mycoses, no. (%)						Prevalence rate ratio† (95% CI)	
		Any mention			Underlying cause			Any mention	
		All	Pulmonary		All	Pulmonary		All	Pulmonary‡
Silicosis	6,723§	48 (0.71)	37 (0.55)		25 (52)	21 (57)		4.5 (3.4–6.0)	9.5 (6.9–13.1)
Asbestosis	23,899§	58 (0.24)	46 (0.19)		22 (38)	18 (39)		1.5 (1.2–2.0)	3.3 (2.5–4.4)
CWP	46,088§	72 (0.16)	38 (0.08)		29 (40)	16 (42)		1.0 (0.8–1.2)	1.4 (1.0–2.0)
All other deaths¶	51,677,216	81,699 (0.16)	29,914 (0.06)		33,941 (42)	12,982 (43)		NA	NA

*Data from National Center for Health Statistics (www.cdc.gov/nchs/products/elec_prods/subject/mortmcd.htm). CI, confidence interval; CWP, coal worker pneumoconiosis; NA, not applicable. †Proportion of decedents with each selected pneumoconiosis who had mycosis coded as the underlying cause of death or as a contributing cause of death on the entity axis compared with the analogous proportion of pulmonary mycosis decedents without any mention of a selected pneumoconiosis as underlying cause of death or a contributing cause of death on the entity axis (all other deaths). ‡When International Classification of Diseases (ICD) 9th or 10th revision codes for opportunistic mycoses and ICD-10 codes for unspecified types of mycoses were excluded, the prevalence rate ratio and 95% CI for deaths with any mention of silicosis became 10.4 (7.5–14.4); asbestosis 3.3 (2.4–4.4); and CWP 1.5 (1.1–2.1). §No. persons who died for which the selected pneumoconiosis was coded as the underlying cause of death or as a contributing cause of death on the entity axis, excluding decedents with multiple pneumoconioses. ¶Excludes deaths with any mention (underlying or contributing cause) of silicosis, asbestosis, and/or CWP.

Among persons who died with pneumoconiosis, aspergillosis was the most common pulmonary mycosis. Those with silicosis were more likely than those without any pneumoconiosis to have aspergillosis, coccidioidomycosis, or cryptococcosis at death (Table 2). Among the 48 decedents who had silicosis and mycosis, 9 also had tuberculosis, 4 had diabetes (2 with tuberculosis), 2 had lung malignancy (1 with tuberculosis), and none had received organ transplants. From 1987 (the first year of ICD codes for HIV) to 2004, 1 decedent with silicosis had HIV but not mycosis. Of 8 decedents with asbestosis and HIV, 2 had mycoses; no decedents with CWP had HIV. When we limited analysis to decedents in the southwestern states (Arizona, California, Nevada, New Mexico, Texas, and Utah), those with silicosis were 27.9× (95% CI 12.6–62.0×) more likely to have coccidioidomycosis at death than were decedents without pneumoconioses, and those with asbestosis were 4.5× (95% CI 1.9–10.8×) more likely to have coccidioidomycosis at death than were decedents without pneumoconioses. Decedents with silicosis from states bordering the Ohio, Missouri, and Mississippi River valleys, were 4.8× (95% CI 0.7–34.0×) more likely to have histoplasmosis at death than were decedents without pneumoconiosis, and decedents with asbestosis were 3.9× (95% CI 1.3–12.2×) more likely to have histoplasmosis than were decedents without pneumonconioses.

**Table 2:** Cause of death for decedents with any death certificate mention of selected pneumoconioses (underlying or contributing cause) and any mention of the most common types of pulmonary mycosis, US residents ≥45 y of age, 1979–2004*

Cause	Pulmonary mycosis, prevalence rate ratio (95% CI)			
	Coccidioidomycosis†	Histoplasmosis‡	Aspergillosis§	Cryptococcosis¶
Silicosis	17.9 (8.0–39.9)	2.9 (0.4–20.7)	13.9 (9.2 – 21.1)	6.9 (3.3 –14.5)
Asbestosis	4.2 (1.8–10.1)	3.3 (1.2–8.8)	5.5 (3.9– 7.8)	0.8 (0.3–2.6)
CWP	0	0.4 (0.1–3.0)	1.8 (1.2–2.9)	0.9 (0.4–1.9)

*Compared with persons who died without any mention of selected pneumoconioses. Data from National Center for Health Statistics (www.cdc.gov/nchs/products/elec_prods/subject/mortmcd.htm). CI, confidence interval; CWP, coal worker pneumoconiosis. †Six with coccidiodomycosis among 6,723 decedents with silicosis; 5 among 23,899 decedents with asbestosis; zero among 46,088 with CWP; 2,576 among 51,677,216 without silicosis, asbestosis, and/or CWP. ‡One with histoplasmosis among 6,723 decedents with silicosis; 4 among 23,899 with asbestosis; 1 among 46,088 with CWP; 2,633 among 51,677,216 without silicosis, asbestosis, and/or CWP. §Twenty-two with aspergillosis among 6,723 decedents with silicosis; 31 among 23,899 with asbestosis; 20 among 46,088 with CWP; 12,165 among 51,677,216 without silicosis, asbestosis, and/or CWP. ¶Seven with cryptococcosis among 6,723 decedents with silicosis; 3 among 23,899 with asbestosis; 6 among 46,088 with CWP; 7,786 among 51,677,216 without silicosis, asbestosis, and/or CWP.

## Conclusions

We found that persons who die with silicosis are more likely to die with pulmonary mycosis than are those who die without pneumoconiosis or who die with the more common pneumoconioses. Insofar as silica dust impairs cellular defense, silica-exposed workers (without silicosis) may be at increased risk for fungal infections, as they are for mycobacterial infections (*1*).

Aspergillosis was the most common mycosis among persons with pneumoconiosis. Aspergillosis is a known complication in patients with underlying pulmonary disease, such as pulmonary tuberculosis and pneumoconiosis (*2*), in which silica-impaired macrophages are incapable of targeting inhaled conidia (*3*). The rarity of candidiasis in persons with silicosis may reflect the fact that healthy workers are less likely to have concurrent diabetes or HIV infection.

Concurrent mycosis was specific to decedents with silicosis compared with those with asbestosis or CWP, possibly because coal mine dust and asbestos fibers are less toxic to macrophages than are crystalline silica. Persons with asbestosis and CWP are also less commonly affected by autoimmune diseases and systemic immunologic complications than are those with silicosis (*4**,**5*).

Direct impairment of macrophage function by crystalline silica and poor drug penetration into silicotic lung nodules have resulted in high (>20%) treatment failure and relapse rates for patients with silicosis who are receiving chemotherapy for tuberculosis (*6*). This finding has prompted prolonged and more aggressive treatment of tuberculosis for such persons (*7*). Similarly, treatment of mycosis in patients with silicosis or substantial past exposure to silica dust may require prolonged treatment and possibly chronic suppressive antifungal therapy, as is used for patients with immunocompromised conditions (*8*).

Lacking population-based surveillance data for silicosis and mycosis illness and silica exposures, we relied on death certificate data, which have limitations; e.g., only ≈1 of 6 persons who had silicosis had silicosis recorded as a cause of death on the death certificate (*9*). Also, many persons with substantial exposure to silica dust never receive a diagnosis of silicosis. We were unable to address the question of possible increased risk for mycosis among silica-exposed persons, and our analysis may underrepresent the actual extent of concurrent silicosis and mycosis. Another limitation was use of ICD-9 and ICD-10 coding for fungal infections and for fungal infection causing death. Sensitivity of this method can vary at different institutions and over time, especially if fungal infections are underdiagnosed. In addition, the ICD-9 classification codes for many mycoses do not differentiate pulmonary from other types of mycoses.

Health professionals should consider enhanced risk for mycosis with regard to preventive interventions, differential diagnosis, and mycosis treatment of silica-exposed workers. Measures to protect silica-exposed workers with coexposure to fungi include reducing silica exposure; wetting soil and bird droppings to suppress fungal-contaminated dust; maintaining good personal hygiene; and, in areas with endemic inhaled fungi, using enclosed operator cabs with high-efficiency particulate air filtration or personal respiratory protection for particulates (*10*).
